# Supplementary material for: Plant-Growth-Promoting Potential of PGPE Isolated from Dactylis glomerata L
Source: Microorganisms. 2022 Mar 29;10(4):731. doi: 10.3390/microorganisms10040731 (PMC9032031; doi:10.3390/microorganisms10040731)
Supplement: Supplementary file 1 [file microorganisms-10-00731-s001.zip › microorganisms-1647483-supplementary.pdf]

Supplementary Material

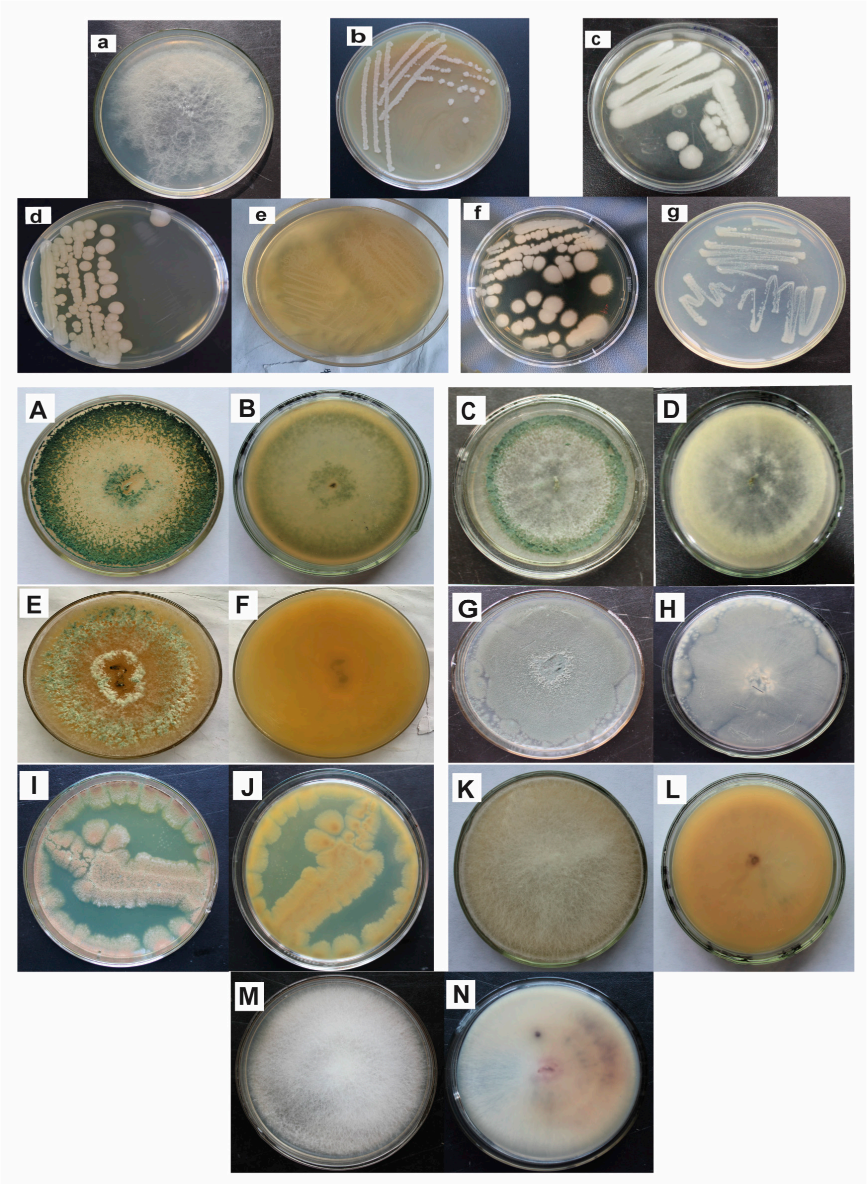

Figure S1. culture morphology of endophytic bacteria and fungi in *D. glomerata*.

Notes: a represents 344G1-B, b represents 344J1-B, c represents 586G1-B, d represents CFY1-B, e represents TG1-B, f represents TJ1-B, g represents TG2-B. A represents the front side of 344G1-F medium, B represents the opposite side of 344G1-F, C and D represent the front and back sides of 344G2-F medium respectively, E and F represent the front and back sides of 344G3-F medium respectively, G and H represent the front and back sides of 586G1-F respectively, I and J represent the front and back sides of 586J1-F respectively, K and L represent the front and back sides of TG2-F, M and N represent the front and back sides of TG3-F respectively.
